# Patient and Public Involvement in Action: Shaping an Intergenerational Engagement Intervention in a Long‐Term Care Facility in China

**DOI:** 10.1111/hex.70451

**Published:** 2025-10-01

**Authors:** Hao Liu, Anne Topping, Ping Guo

**Affiliations:** ^1^ Department of Nursing and Midwifery, School of Health Sciences, College of Medicine and Health University of Birmingham Birmingham UK; ^2^ University Hospitals Birmingham NHS Foundation Trust Birmingham UK

**Keywords:** China, health and social care research, intergenerational engagement, intervention, long‐term care facility, patient and public involvement

## Abstract

**Aim:**

To shape an intergenerational engagement (IE) intervention through consultation with a patient and public involvement (PPI) group.

**Design:**

This paper describes how PPI contributed to refining an IE intervention within a long‐term care facility in China.

**Data Sources:**

A PPI group composed of key stakeholders, including older residents of the long‐term care facility, young people from a local school, a parent, and care facility staff, provided evaluative feedback. They reviewed and refined various aspects of the intervention (content, scheduling and delivery of the intergenerational activities) and its evaluation (design, recruitment strategies and study materials).

**Results:**

Key modifications included adjusting activities to accommodate diverse backgrounds and technological capabilities, space limitations and the number of participants to optimise engagement and focusing on group activities to encourage lively and inclusive interactions. The group also made recommendations relating to scheduling sessions, length of intervention and recruitment materials to facilitate greater accessibility.

**Discussion:**

The integration of PPI made the IE intervention practical, culturally relevant and tailored to meet the diverse needs of older and younger participants, enhancing its feasibility, acceptability and overall effectiveness. The findings underscore the importance of early and continuous PPI in health and social care research to develop relevant and sustainable interventions. Future efforts should focus on policy support, funding and education to embed PPI more broadly into health and social care research in China.

**Patient or Public Contribution:**

Feedback was sought from a patient and public involvement (PPI) group to refine and enhance the intergenerational engagement (IE) intervention, ensuring it accurately reflected the needs of participants. Notably, to the best of the authors' knowledge, this study is the first in China to utilise PPI in the co‐design of an IE intervention in a long‐term care setting, offering valuable insights for future research and practice in this context.

## 1 Introduction

### 1.1 PPI in Health and Social Care Research

Patient and public involvement (PPI) is widely recognised as best practice in health and social care research, engaging patients, carers and the public as partners in research design, delivery and dissemination [1, 2]. Many Western countries, such as the United Kingdom, the United States, Canada, Australia, and those in Europe, have embedded PPI into national health research frameworks and funding structures to ensure that research addresses stakeholder needs and priorities [3, 4, 5, 6, 7]. Recent research also highlights practical methods for PPI, including collaboration with existing patient support groups, formation of advisory panels, and the use of online patient panels, all of which can facilitate meaningful engagement and enrich the research process [8].

### 1.2 Challenges and Opportunities for PPI in China

Although PPI is well‐established in Western health and care research, it remains in its early stages in many Asian countries and regions [9]. For example, in Singapore and Hong Kong, efforts to involve the public are only recently emerging through community‐based activities and the gradual introduction of patient perspectives in research [10, 11]. In mainland China, PPI is still in its infancy. In 2020, only 3.4% of BMJ Open papers from China included PPI, compared to 44.5% from the United Kingdom [12], highlighting a significant gap in the integration of PPI into research.

One important reason for this difference is that, unlike Western countries, PPI requirements in academic publishing have not yet been widely adopted in China. Several international journals, such as the British Medical Journal (BMJ), Health Expectations and the Journal of Advanced Nursing, now require authors to include PPI statements in their submissions [13, 14, 15]. However, Chinese journals such as the South China Journal of Preventive Medicine and the Journal of Nursing (China) do not yet have this requirement [16, 17]. This gap arises from limited academic publishing requirements for PPI in China and cultural factors where traditional norms favour expert‐led approaches, resulting in stakeholder participation in research often being limited or overlooked [18]. Local efforts, such as small patient advocacy groups, some community‐based activities, and occasional public representation in research ethics committees, are developing but not yet widespread [19]. The lack of established frameworks and institutional support further limits the integration of PPI into health and social care research in China [19].

Recent research indicates the potential feasibility of locally adapted PPI in China, as demonstrated by collaborative decision‐making and co‐design initiatives involving patients and clinicians in hospital settings [20, 21]. However, little is known about PPI implementation outside the hospital context. Unlike previous studies that have focused primarily on Chinese hospital settings, this study demonstrates the application of PPI in the co‐design of an intergenerational engagement (IE) intervention in a Chinese long‐term care facility. This provides valuable insights into applying PPI in Chinese elderly care, offering a practical example for refining complex interventions.

### 1.3 Ageing in China

China is experiencing rapid demographic ageing, with the proportion of people aged 60 and above rising from 13.3% in 2010 to 18.7% in 2020, totalling 264.02 million people, including 190.64 million aged 65 and older [22]. This demographic shift, driven by declining fertility rates and increased life expectancy, has led to more older people residing in care institutions rather than with their families, raising challenges related to social isolation and diminished quality of life among older people [23, 24, 25]. These trends highlight the need for approaches that support the mental and social well‐being of older people by fostering meaningful interaction and social connection.

### 1.4 IE

IE has been described in the literature as involving organised interactions between older and younger generations to promote mutual understanding, respect and reciprocal learning [26, 27, 28]. In practice, IE is often delivered as an intervention designed to facilitate interaction. Hence, the terms ‘IE intervention’ and ‘IE interaction’ are used interchangeably. Previous studies have shown that IE can effectively address social isolation and loneliness among older people, improving their emotional and social well‐being [29, 30]. To maximise effectiveness, IE interventions should be adapted to local cultural and developmental needs and offer a variety of activities to meet diverse interests [28, 31].

However, our recent systematic review found that several IE studies aimed at enhancing the health and well‐being of older residents in long‐term care facilities did not incorporate PPI in intervention development or design [32]. Other research further emphasised the need for greater involvement of all participant groups in IE to bridge the gap between best practices and improved outcomes [28]. Evidence indicates early input from stakeholders improves intervention relevance, effectiveness and sustainability [33]. To the best of the authors' knowledge, this study is the first in China to utilise PPI in the design of an IE intervention in a long‐term care facility, offering valuable insights for future PPI research in elderly care within the Chinese context.

## 2 Materials and Methods

### 2.1 Study Setting

Guangzhou, the capital of Guangdong Province, is experiencing rapid population ageing: by the end of 2023, 19.4% of its 10.57 million residents were aged 60 or above and 13.9% were aged 65 or above [34]. Rising numbers of older people living alone have further increased demand for institutional care [35]. Our study took place in an urban long‐term care facility that provides integrated medical and nursing care (Yi Yang Jie He^1^), alongside rehabilitation services and a variety of amenities. During our study period, 131 beds were occupied out of 162 available beds, including 27 reserved for residents requiring full assistance with daily activities.

### 2.2 PPI Group Formation and Recruitment

We recruited a small PPI group to co‐design and refine an IE intervention that would be evaluated through a feasibility study. Due to the limited space of the activity room in the facility, our feasibility study was capped at 50 participants. Accordingly, the PPI group was intentionally kept small to facilitate detailed, iterative discussions aligned with the scale of the feasibility study. Purposive sampling was used to include perspectives across age, gender, experience and roles. Six members were recruited: one staff member from the long‐term care facility, two older residents, two young people from a local school and one parent. Group composition details are shown in Table 1. Whilst the group was not designed to be representative, it included members from the different stakeholders. This was considered important to ensure that the planned intervention reflected the diverse needs and perspectives of the target population.

**Table 1:** Details about the PPI group.

Code^a^	Gender	Age	Education level	Background	Inclusion criteria	Contributions
S1	Female	28	Above high school	Nurse and serves as an associate manager Has some research experience	a. Willingness to participate and time capacity b. Ability to collaborate c. Knowledge of health and social care research (prioritised if applicable)	‐ Provide insight into the delivery of social activities in the long‐term care facility ‐ Provide insight into logistics and implementation ‐ Identify the environment for IE to maximise access for residents with limited mobility issues ‐ Assist in the recruitment of residents
O1	Male	68	Above high school	Children and grandchildren live abroad Held a managerial position in a company before retirement	a. Resident in a long‐term care facility b. Willingness to participate c. Able to read and provide feedback d. No cognitive impairment^b^	‐ Provide insight into the capabilities, interests and motivation of residents to participate in IE ‐ Offer feedback on IE intervention design duration, frequency and types of activities ‐ Review questionnaires and topic guides for use in data collection ‐ Provide insight into the recruitment of residents
O2	Female	71	Below high school	Children and grandchildren live in the same city Did not have a formal job		
Y1	Female	16	High school	Had experience participating in IE activities in the community	a. Willingness to participate and time capacity b. Communication skills c. Relatively good physical and mental condition^c^	‐ Offer a critical review of the proposed IE intervention ‐ Suggest interactive and dynamic activities that would foster engagement of young people with residents ‐ Assist in the recruitment of peer participants
Y2	Male	16	High school	Lived with grandparents		
P1	Female	42	Above high school	Mother of two children, a son and a daughter Majored in law	a. Willingness to participate and time capacity b. Supportive attitude and effective communication	‐ Review processes for obtaining parental informed consent and young people's assent, ensuring ethical compliance

^a^ S, staff; O, Older resident; Y, Young people; P, Parent.

^b^ Cognitive ability for O1 and O2 was assessed by reviewing their medical records and regular health assessments conducted by the doctor at the facility. S1 also confirmed their eligibility through informal daily interactions and brief conversational screening.

^c^ ‘Relatively good physical and mental condition’ is defined as the absence of acute illness or psychological disorders, as well as the ability to participate fully and communicate effectively in PPI activities. According to national guidelines, schools need to maintain annual health records for every student, which teachers can access alongside classroom observations. This enables teachers to identify and nominate students with consistently good physical and mental health. Retrieved from http://www.moe.gov.cn/jyb_xxgk/moe_1777/moe_1779/202110/t20211027_575478.html.

However, several challenges emerged during the process. Many lacked awareness and understanding of PPI, expressing uncertainty about its purpose and how their input would shape the intervention. Some doubted the value of their lived experience, feeling unqualified due to a lack of research experience. To address these challenges, the recruitment process began by engaging a staff member (S1) with a medical background and research experience. The concept, purpose and value of PPI were first explained to her in detail. Drawing on her established rapport and familiarity with the residents, S1 identified two older residents (O1 and O2) whom she considered suitable and who met the inclusion criteria. Her involvement was instrumental in bridging the gap between the research team and the older residents, as she helped the researchers build relationships and trust, address concerns, and encourage participation.

**Figure 1:**
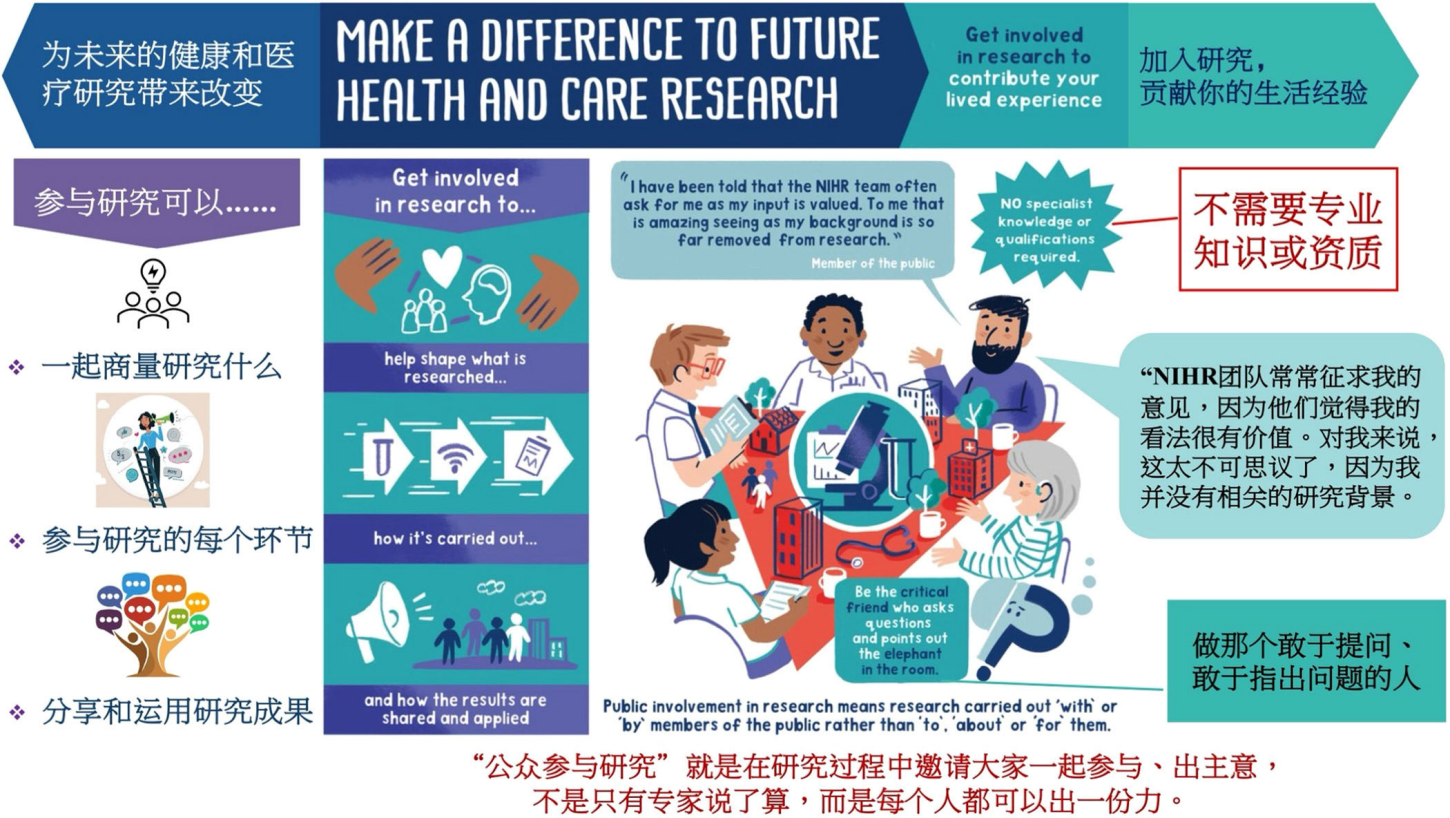
Chinese translation and adaptation of the NIHR infographic.

A local teacher provided essential support for the recruitment of young people. Although the teacher did not have any research experience, she had a high level of education. To facilitate her understanding of PPI, she was provided with relevant references and supporting materials. Once she was familiar with the concept and aims of PPI, she recommended two suitable young people (Y1 and Y2) to join the group.^2^ In addition, she helped contact the head of the parent committee^3^ (P1), who was then included as the parent representative to provide legal and ethical perspectives, particularly regarding consent for young people's participation. Since most stakeholders had limited research experience, the importance and benefits of PPI were explained in clear, accessible language, emphasising how their lived experiences could help shape the intervention [2]. Plain language and minimal jargon were used to encourage broader involvement and diverse perspectives [36]. The references and materials provided during recruitment are summarised in Table 2, with Figure 1 illustrating the adapted National Institute for Health and Care Research (NIHR) infographic.

**Table 2:** Sources and adaptations for explaining PPI concepts to stakeholders.

Sources	Type	Key content to use	Notes
Public and patient involvement in UK Medical Research (Original title: 英国公众与患者参与医学研究的实践)^a^	Chinese Journal	Presents the principles and value of PPI, explains the reasons for public involvement, diverse participation methods, typical case studies from the United Kingdom, and benefits for improving health research and services	Reduce medical jargon and technical terms while using plain language to make PPI concepts accessible to stakeholders
Research Progress on Patient and Public Involvement in the Development of Clinical Practice Guidelines and Its Methods (Original title: 患者及公众参与临床实践指南构建及其方法的研究进展)^b^	Chinese Journal	Explains what PPI is, how it differs from the traditional research model and provides details on who can participate, how they are involved, and the practical steps for effective PPI in guideline development	
NIHR public involvement infographic^c^	Infographic	Clearly and visually conveys the benefits and steps of PPI, making the information accessible and easy to understand	Provide the infographic alongside a simple Chinese translation for easy understanding (see Figure 1)
Principle 1: Involve the right people––Who are the right people?^d^	Webpage	Emphasises the value of involving people with relevant lived experience and how their insights can guide and enhance the research process	Translate into Chinese and use plain language to make it accessible for stakeholders
Patient and public involvement (PPI) in healthcare teaching^e^	Video	Provides a few examples of how PPI contributes to learning experiences and benefit from sharing their stories	Provided in original English, as staff, teacher and young people have sufficient English proficiency. The researcher also provided explanations when the video was being viewed

^a^ Ji, P., and Chu, H. L. (2020). Public and Patient Involvement in UK Medical Research (in Chinese). *Chinese Journal of Medical Research Management* (20), 18–22.

^b^ Zhou, F. (2020). Research Progress on Patient and Public Involvement in the Development of Clinical Practice Guidelines and Its Methods (in Chinese). *Chinese Journal of Nursing*, *27*(5), 14–17.

^c^ Starting out guide—why and how to get involved in research. NIHR. Retrieved from https://www.nihr.ac.uk/starting-out-guide-why-and-how-get-involved-research.

^d^ Principle 1: Involve the right people. Health Research Authority. Retrieved from https://www.hra.nhs.uk/planning-and-improving-research/best-practice/public-involvement/principle-1-involve-right-people/.

^e^ Video from Cardiff University. Retrieved from https://www.youtube.com/watch?v=I2-veOQoR88.

### 2.3 Process of PPI Activities

Following the guidance provided by the Medical Research Council (MRC) framework for developing and evaluating complex interventions [37], our research process began with a systematic review to identify key components of IE interventions, evaluation measurement tools, research gaps, and uncertainties. Key uncertainties included whether older residents and young people would be willing to participate, whether the intervention was logistically viable, whether parents would consent to their children's involvement, whether the evaluation scales were appropriate, and whether IE had a meaningful impact on stakeholders. The updated MRC framework outlines four phases with core elements, emphasising stakeholder collaboration to refine research questions, design studies and apply appropriate methodologies in research [37]. Integrating PPI into the intervention refinement process ensured that the research was relevant, feasible and acceptable to participants and facilitated dissemination of findings to a wider audience [33].

The PPI group was formed during the refinement process of the IE intervention in February 2024 and was formally established in early March 2024. Research suggests that to sustain ongoing participation from stakeholders, flexible and strategic communication methods should be implemented [38]. To sustain participation and foster continuous communication, a WeChat^4^ group was established. The draft design of the IE intervention was developed based on insights from a systematic review and guided by the activity theory of ageing [39]. The draft was then presented to the PPI group, along with all related materials, for their review and feedback. Through clear and focused discussions, PPI members were encouraged to review and refine various aspects of the IE intervention. This included the content, scheduling and delivery of intergenerational activities, as well as identifying potential barriers that could hinder participation from both older residents and young people. They also contributed to refining questionnaires, recruitment strategies and other study materials.

The PPI activities commenced with an initial online meeting involving all PPI members, allowing broad perspectives and collective feedback on the overall design, feasibility and relevance of the IE intervention. To mitigate potential power imbalances, the meeting began with explicit ground rules that emphasised inclusive participation and the equal value of all contributions. The focus was on eliciting insights from all PPI members, rather than engaging in debate, to ensure that each voice was heard and respected. Rather than seeking consensus, the PPI members aimed to incorporate a diverse range of viewpoints to maximise practicality [40]. Follow‐up meetings were then held separately with older residents, young people and staff to ensure balanced engagement. These smaller sessions created safe spaces for each group to express ideas and concerns without hierarchical pressure. Continuous feedback was also encouraged through WeChat, enabling members to share their thoughts at any time.

**Figure 2:**
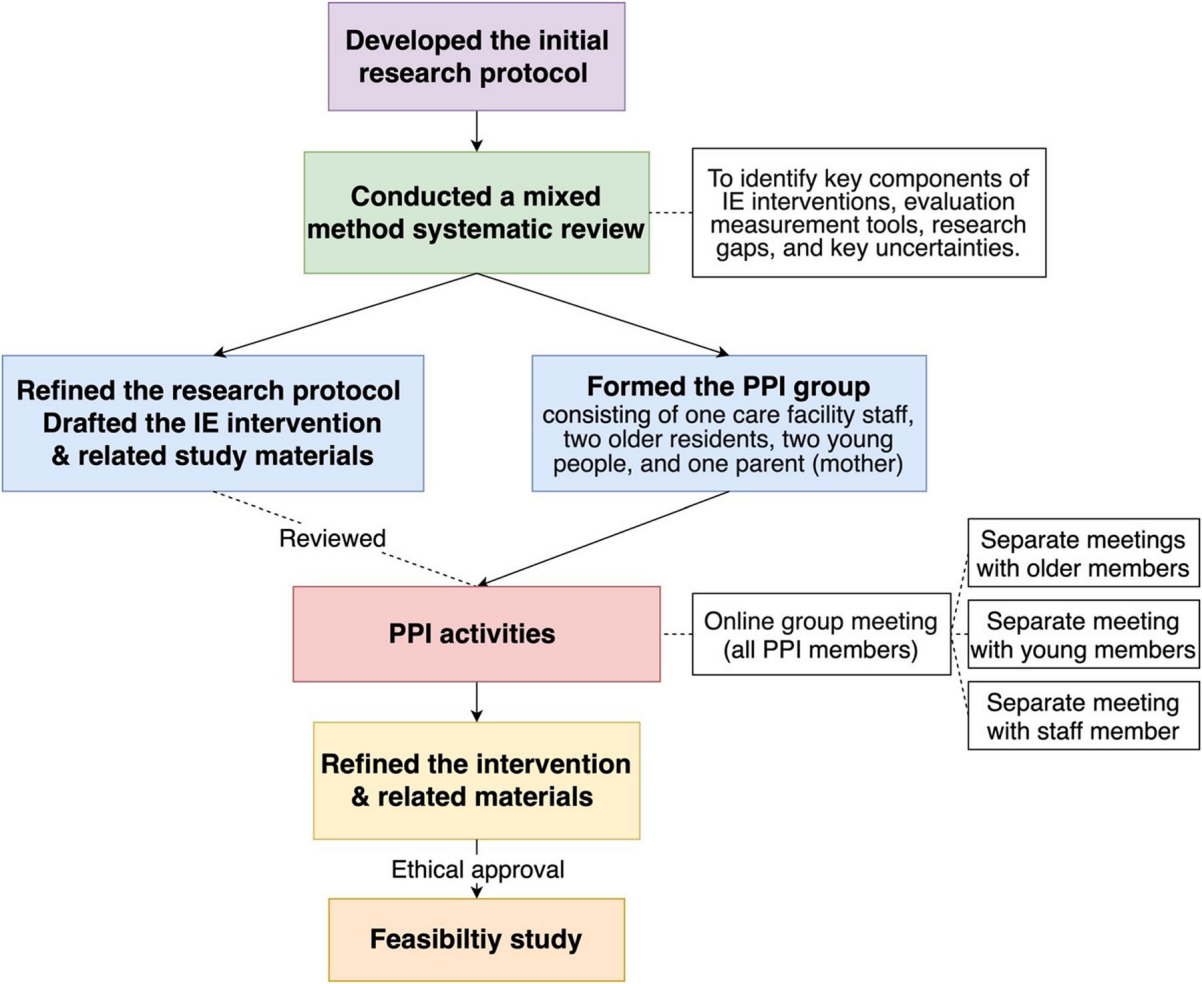
Process of shaping the IE intervention through PPI.

Within these meetings, each subgroup focused on areas relevant to their experience. Older PPI members focused on reviewing the questionnaire, as older participants in the feasibility study would be required to complete it before and after the intervention. The aim was to assess its clarity, accessibility and readability for the target audience. Before the meeting, they piloted the questionnaire, which included demographic information and four validated scales in Chinese to assess depression, anxiety, loneliness and quality of life [41, 42, 43, 44, 45]. Young PPI members discussed recruitment, including how to attract their peers, effectively introduce the intervention and encourage participation through peer networks. The staff member discussed practical implementation, addressing recruitment challenges, logistical arrangements and emergency planning to ensure smooth delivery of the intervention. The process of shaping the IE intervention through PPI is shown in Figure 2, which outlines key stages from initial protocol development to the feasibility study.

Based on the feedback, several refinements were made to improve the IE intervention and supporting materials. Refinements included modifying activities to better align with participant needs, adjusting the schedule to enhance feasibility and revising key documents such as participant information sheets and consent/assent forms to improve clarity and readability. By incorporating stakeholder input during the revision period before applying for ethical approval, the IE intervention was refined to be relevant, feasible, acceptable and practical. PPI activities do not require formal ethical approval, but increasingly there is an expectation that researchers make efforts to consult with stakeholders before review by institutional ethical committees. This study was reviewed and approved by the University of Birmingham Research Ethics Committee (Ref: ERN_1775‐Jul2024). The feasibility study was also registered with the ISRCTN registry (ISRCTN14922432). The adopted PPI process strengthened the ethical integrity of the research by ensuring that the proposed intervention aligns with participant needs and ethical principles.

## 3 Results

### 3.1 Overview of the IE Intervention

The IE intervention was seen as a positive approach for improving recreational activities in the long‐term care facility while fostering intergenerational connections and meeting the social interaction needs of older residents.

### 3.2 Participant Number and Space Capacity

Due to the limited size of the activity room in the long‐term care facility, it was suggested to restrict the number of participants to avoid excessive noise, which could negatively affect the effectiveness of the activities, be challenging for those with impaired hearing and disturb other residents not participating in the IE intervention.

### 3.3 Group Versus One‐on‐One Activities

Through a co‐design process, PPI members shaped the format of IE activities. Older and younger members considered one‐on‐one interactions potentially monotonous and less dynamic, especially during initial interactions when participants were still unfamiliar with each other. The staff member also noted group activities as preferable for fostering a lively atmosphere and enhancing active participation among all participants in the feasibility study.

### 3.4 Suggested Warming Up Activities

Warm‐up activities were suggested to create a lively environment, break the ice among participants, encourage participation, and facilitate interpersonal connections. Older PPI members prioritised simple activities, young members suggested interactive games and quizzes to enhance engagement, the staff member emphasised practical options suitable for group cohesion within the facility, and the parent highlighted activities making young participants comfortable and actively involved. These suggestions reflect the PPI group's intergenerational perspectives. Table 3 summarises these warm‐up activity suggestions.

**Table 3:** Warm‐up activities recommended by PPI members.

Warm‐up activity	Suggested by	Intergenerational perspective
Clapping exercises	Older members, staff	Simple, inclusive and suitable for varying mobility Encourages group participation and is easily integrated for all ages
Fun quizzes	Young members, staff, parents	Stimulates memory and learning Fosters friendly competition and teamwork Appeals to both generations and encourages collaborative engagement
Games (e.g., board games, card games, music games and outdoor games)	Older and young members, staff	Offers a variety of options to match different interests and abilities Creates a lively atmosphere and promotes interaction across age groups
Comic skits/crosstalk	Young members	Provides humour and entertainment Facilitates initial interaction and encourages participation

### 3.5 Modifications to Sessions

PPI members collaboratively reviewed and refined session content and structure. Some activities were simplified, as overly complex tasks could become overwhelming or frustrating, particularly for older residents. Simplified activities ensured inclusivity, enabling all participants to engage effectively. One session initially titled ‘The Beauty of Cantonese Opera’ was changed to ‘The Beauty of Classic Songs’. While Cantonese opera is culturally significant, it was recognised that it might be less familiar to participants from different regions or generations. Classic Chinese songs provided a broader musical variety, facilitating greater intergenerational connection and improved accessibility. Another planned session originally focused on calligraphy, but this was changed to painting after PPI members observed that calligraphy was less accessible and potentially challenging for older residents. Painting was viewed as a more suitable option, allowing for greater participation and enjoyment among all generations. In another session, ‘Past and Present’, it was initially planned that participants would create digital memory books using smartphone apps. Recognising that many older residents were unfamiliar with advanced smartphone features beyond basic communication and WeChat, the activity was adapted. Instead, young participants took photos and created pictures with a fashion theme, making the activity more engaging and accessible for older residents. This adjustment helped bridge the generational gap and ensured participation regardless of technological familiarity and digital literacy.

### 3.6 Scheduling Considerations and Session Structure

The long‐term care facility typically does not organise weekend activities, as residents often spend time with their families or attend to personal matters. However, weekday scheduling conflicted with young people's school timetables, which presented a challenge. Consequently, the summer school holiday was identified as the optimal period for the IE intervention, as both groups would be more available. The intervention was originally planned as a 6‐week programme but was ultimately adjusted to 5 weeks to fit the duration of the summer holiday of young people. Sessions were scheduled to start at approximately 3:20 p.m., aligning with the facility's daily routine, as many older residents typically rest until 3 p.m.^5^

Weekly sessions were considered, with a suggestion to extend the duration from 60 to 90 min. This extended time frame allowed for more meaningful engagement, enabling participants to fully enjoy and participate in activities, enriching the overall experience and strengthening intergenerational connections. However, older members and staff raised concerns that longer sessions might lead to fatigue or reduced attention towards the end of the activity. Therefore, breaks were incorporated, and activities were paced as needed to help maintain the comfort and engagement of older residents throughout. The main components of the IE intervention and the changes co‐designed with PPI members are shown in Table 4.

**Table 4:** Components of the IE intervention co‐designed with PPI members.

IE component	Initial plan	Co‐designed changes	Suggested by	Rationale
Activity format	Not specified	Group activities	All members	Group activities seen as more dynamic and inclusive, especially during initial sessions
Warm‐up activities	Not specified	Introduced warm‐up activities (e.g., quizzes, clapping and games)	All members	Facilitate ice‐breaking, encourage engagement and support interaction across generations
Session modification	Cantonese Opera	Classic Chinese songs	Older and young members, staff	Classic songs are more familiar and engaging for both young people and older residents
	Calligraphy	Painting	Older and young members, staff	Painting is more accessible for older residents and allows for broader participation
	Create a digital memory book via apps	Young people take photos and create fashion‐inspired images	Older and young members, staff	Adapted for older residents less familiar with advanced smartphones or apps, ensuring engagement.
Scheduling and duration	6 weeks	Adjusted to 5 weeks, aligned with the summer holiday	Young members	Matches young people's availability
	60‐min sessions	90‐min sessions with scheduled breaks	Older members, staff	Allows for deeper engagement while accommodating older residents' comfort and rest

### 3.7 Review of Relevant Study Materials

#### 3.7.1 Leaflet Enhancements

The leaflet describing the IE intervention was considered clear and effective. However, suggestions for improvement included explicitly stating session frequency (weekly) and duration (90 min), adding contact information for inquiries, and increasing font size to enhance readability for older residents. The leaflet complemented participant information sheets, clearly outlining session agendas and supporting recruitment.

### 3.8 Information Sheets and Consent/Assent Forms

PPI members found the participant information sheets and consent/assent forms clear and easy to understand, but recommended enhancements to improve accessibility for the different target groups. Older members suggested increasing the font size for better readability, simplifying language to avoid confusion, and adding contact information for clarification. Younger members proposed rewording sections to better match their reading habits and improve readability. The parent recommended emphasising the potential benefits to the young people gained from participation in the intervention. These suggestions aimed to make the materials more inclusive and user‐friendly for all intended audiences.

### 3.9 Questionnaire

After completing the questionnaires, older members found the questions clear and easy to understand, taking approximately 40 min to complete. The straightforward wording enabled them to answer without requiring additional explanations or assistance. The completion time was considered reasonable, as it matched the estimated duration stated in the information sheet and allowed them to finish without feeling rushed or fatigued. The staff member provided feedback on the demographic information section, suggesting that the religion options be simplified to ‘None’, ‘Have (please specify)’ and ‘Prefer not to say’, reducing cognitive load. Staff also recommended administering the pre‐test questionnaire 1 day before the IE intervention to avoid delays or forgetfulness among older residents.

## 4 Discussion

The integration of PPI in the refinement of this IE intervention revealed several key insights and necessary improvements to enhance effectiveness. Active participation by PPI members was instrumental in shaping an intervention that was both practical and culturally relevant. This section discusses the implications, implementation challenges, and significance for future research and practice in China.

### 4.1 Significance of PPI in the Intervention Design

The importance of PPI in designing the intervention cannot be overstated. Engaging stakeholders incorporates different perspectives and lived experiences, making the intervention more relevant and effective [46]. This is particularly crucial in an IE intervention involving older and younger participants, each with unique needs and expectations [47]. Carman's research emphasises that involving patients and families in the design and implementation of health interventions significantly improves their ability to meet participants' needs [48]. By incorporating input from PPI members, the IE intervention was better tailored to foster meaningful intergenerational friendships and address practical concerns.

Empirical evidence further supports the role of PPI in shaping meaningful interventions. An exploratory study involving care home residents with cognitive and communication impairments found that stakeholder feedback influenced research outcomes and led to unexpected improvements in care home practices [49]. Well‐conducted PPI helps support public interests, ensures research remains relevant with clear outcomes and contributes to practical benefits such as reducing waste and improving research quality [50]. In our study, co‐design processes allowed PPI members to collaboratively refine activities, ensuring cultural appropriateness and feasibility.

### 4.2 PPI Enhancing the Feasibility, Acceptability and Implementation of Interventions

Beyond shaping intervention design, the PPI group significantly contributed to feasibility, acceptability and practical implementation. Active collaboration and continuous feedback from PPI members helped refine the intervention to better address the needs and preferences of all involved. This aligns with Franklin's study, which highlights core PPI values such as representativeness, transparency, accessibility, responsiveness, accountability and sustainability [51]. The positive impact of PPI on intervention acceptability and feasibility has been demonstrated widely. Doughty's study on HIV testing incorporated PPI to refine intervention development, study materials and training sessions, ensuring that the intervention was more relevant and appropriate for dental patients and individuals with HIV [52].

Evidence suggests that involving PPI in the early stages of intervention development makes interventions more likely to be considered acceptable, engaging, feasible and effective during formal feasibility studies [53]. Similarly, Gray‐Burrows's study demonstrates that PPI contributes at different stages of research. It helps assess study design acceptability in the planning phase, supports feasibility and sustainability during implementation, and provides insights to enhance participant engagement in dissemination [54]. In this study, establishing the PPI group early enabled ongoing collaborative input, addressing practical considerations such as session timing, accessibility and logistical arrangements, thus ensuring that the IE intervention was both culturally relevant and logistically feasible.

### 4.3 Addressing Challenges in PPI Implementation

Despite the demonstrated benefits, several challenges hindered PPI implementation, especially limited awareness and understanding of PPI among stakeholders. Previous research identifies a widespread lack of awareness regarding the significance, benefits and opportunities of PPI, which remains a major barrier to its adoption in China [19]. This challenge impedes recruitment and meaningful engagement, reducing the effectiveness of PPI. To address this issue, we used clear, simple language and minimised medical jargon when introducing PPI, as recommended by previous research [36], ensuring they could easily understand its purpose and process.

Another challenge stemmed from stakeholders' concerns about their ability to provide meaningful input, as many felt they lacked the expertise or research experiences to contribute effectively. Studies have shown that patients and the public may experience low self‐confidence and frustration about their perceived lack of knowledge in PPI activities [55]. However, the lived experiences of stakeholders are vital, offering unique insights that shape intervention design and implementation [2, 56]. Future research could support stakeholders through workshops, case study examples or interactive materials that demonstrate the value of lived experiences regardless of formal research expertise, thereby building stakeholders' confidence.

### 4.4 Implications for Future Research and Practice

The integration of PPI in refining the intervention underscores the value of involving a range of stakeholders from diverse backgrounds early in the research process. Likewise, it is important to have early and continuous stakeholder engagement in developing health interventions that are both relevant and sustainable [57]. Future research should incorporate PPI to make interventions not only theoretically sound but also practical, relevant and acceptable to the populations they aim to target. Irrespective of the implementation of PPI in China, PPI remains in its early stages, with limited awareness among policymakers, researchers, the public and patients [19].

Cultural factors such as traditional views of medical expertise may also impact the acceptance and adoption of PPI in research. To overcome these barriers, efforts must prioritise raising awareness and fostering broader understanding. Accessible educational resources, diverse communication strategies, and ongoing support can empower meaningful participation in PPI activities [58, 59]. Policymakers and funding bodies should actively promote PPI by developing institutional frameworks, integrating PPI into funding guidelines, and encouraging training programmes in academic and healthcare institutions. Table 5 summarises actionable recommendations from this study for policy and practice to advance the adoption and impact of PPI in China.

**Table 5:** Actionable recommendations for policy and practice to advance the adoption and impact of PPI in China.

Focus area	Recommended actions	Expected impact
Develop policy support for PPI	Establish government policies to support and encourage PPI	Provides a supportive policy environment for the systematic and sustainable implementation of PPI
	Based on these policies, institutions such as universities, hospitals and research organisations should establish specific PPI mechanisms and integrate PPI into research practice	
Integrate PPI into funding and regulation	Integrate requirements for PPI plans in grant applications	Embeds PPI expectations in routine funding and review processes, improving research quality, transparency and reproducibility
	Include PPI criteria in ethical reviews	
	Encourage journals to mandate PPI statements in submissions	
Develop frameworks, guidelines, methodologies and researcher training	Adapt international PPI frameworks to the Chinese context	Provides culturally relevant, practical guidance and builds researchers' capacity for meaningful PPI involvement
	Develop clear, actionable PPI guidelines suited to local practice	
	Provide practical methodologies with step‐by‐step processes and checklists	
	Provide targeted training programmes for researchers on PPI principles and practical skills	
Enhance public education	Develop accessible materials in Chinese (e.g., leaflets, online guides and videos) to educate the public about PPI and its benefits	Raises public awareness and expands the pool of informed members of the public able to contribute to research

### 4.5 Strengths and Limitations

One of the primary strengths of the study used as an example is that, to the best of the authors' knowledge, it is the first to incorporate PPI in an IE intervention within a long‐term care facility in China. The inclusion of a PPI group comprising long‐term care facility staff, older residents, young people and parents brought together a broad range of insights, enhancing acceptability, relevance and practicality of the intervention. The iterative feedback process allowed for continuous improvement and adaptation of the intervention design based on real‐time input from PPI members. However, the study also has limitations. The relatively small and localised nature of the PPI group may not fully represent the varied experiences and needs of broader stakeholders across different regions of China. Furthermore, feedback from the PPI group could be influenced by personal preferences or perspectives, potentially introducing biases that may not fully capture a range of views on the IE intervention. Additionally, it should be noted that the care staff member who assisted with recruitment also joined the PPI group, and this dual role may have introduced potential selection bias.

## 5 Conclusion

This study illustrates how co‐design with PPI members effectively informed the development and refinement of the IE intervention. Active PPI ensured that activities and logistical plans addressed the real‐world needs of both older residents and young people. However, challenges such as limited awareness of PPI, concerns about stakeholder contributions and cultural influences remain, particularly in the context of Chinese research. Future efforts should focus on policy support, funding and education to embed PPI more broadly into health and social care research in China. Addressing these areas may contribute to more inclusive, effective and sustainable health and social care research outcomes in China.

## Author Contributions

**Hao Liu:** conceptualization, methodology, writing – original draft, project administration. **Anne Topping** and **Ping Guo:** supervision, validation, writing – review and editing.

## Conflicts of Interest

The authors declare no conflicts of interest.

## Data Availability

Data sharing is not applicable to this article as no datasets were generated or analysed during the current study.

## Endnotes

1 The Yi Yang Jie He model, which integrates medical treatment with daily care, is currently the predominant model promoted by national policy to address the healthcare needs of China's ageing population. Retrieved from https://www.gov.cn/zhengce/zhengceku/2022-07/22/content_5702161.htm

2 While the teacher assisted with communication and recruitment of young people and a parent, she was not included in the PPI group, as it was limited to those directly participating in the IE intervention. Since she would not be involved in the intervention activities, her input was not required for the design process.

3 P1 was recruited for her position on the parent committee, experience communicating with both teachers and other parents, and her legal background, which provided valuable insight into consent processes. As parents would not directly participate in the intervention but were required to provide informed consent for their children (in accordance with the Law on the Protection of Minors, which defines minors as under 18), her expertise ensured that all ethical and legal requirements for parental consent were fully addressed.

4 WeChat or Weixin in Chinese—an instant messaging, social media, and mobile app developed by Tencent.

5 In Guangzhou, formal lunch breaks run from 12 p.m. to 2:30 p.m. To ensure older residents have sufficient rest before afternoon activities, the facility extends its rest period until 3 p.m.
